# Conformational changes of loops highlight a potential binding site in *Rhodococcus equi* VapB

**DOI:** 10.1107/S2053230X2100738X

**Published:** 2021-07-28

**Authors:** Christina Geerds, Albert Haas, Hartmut H. Niemann

**Affiliations:** a Department of Chemistry, Bielefeld University, Universitaetsstrasse 25, 33615 Bielefeld, Germany; b Institute for Cell Biology, University of Bonn, Ulrich-Haberland-Strasse 61a, 53121 Bonn, Germany

**Keywords:** β-barrels, ligand-binding sites, conformational change, virulence factors, virulence-associated proteins, *Rhodococcus equi*

## Abstract

Virulence-associated proteins (Vaps) contribute to the virulence of the pathogen *Rhodococcus equi*, but their mode of action has remained elusive. All Vaps share a conserved core of about 105 amino acids that folds into a compact eight-stranded antiparallel β-barrel with a unique topology. At the top of the barrel, four loops connect the eight β-strands. Previous Vap structures did not show concave surfaces that might serve as a ligand-binding site. Here, the structure of VapB in a new crystal form was determined at 1.71 Å resolution. The asymmetric unit contains two molecules. In one of them, the loop regions at the top of the barrel adopt a different conformation from other Vap structures. An outward movement of the loops results in the formation of a hydrophobic cavity that might act as a ligand-binding site. This lends further support to the hypothesis that the structural similarity between Vaps and avidins suggests a potential binding function for Vaps.

## Abstract 2

A structure of the *Rhodococcus equi* virulence factor VapB in a new crystal form reveals conformational changes in loop regions at the top of the eight-stranded β-barrel. The resulting cavity might form the binding site for a potential ligand.

## 1. Introduction   

*Rhodococcus equi* is a Gram-positive environmental soil bacterium that can cause severe bronchopneumonia in young foals (Vázquez-Boland & Meijer, 2019 ▸). It also poses a threat to immunocompromised humans such as AIDS patients. *R. equi* infects lung alveolar macrophages, where it multiplies in a remodelled compartment, the *Rhodococcus*-containing vacuole (Zink *et al.*, 1987 ▸; Fernandez-Mora *et al.*, 2005 ▸). This is an unusual phagolysosome that maintains a near-neutral pH (von Bargen *et al.*, 2019 ▸). A virulence-associated plasmid of some 85 kbp is absolutely required for infection of foals (Takai *et al.*, 1991 ▸; Tkachuk-Saad & Prescott, 1991 ▸) and encodes a key factor for infection and intracellular multiplication: virulence-associated protein A (VapA; Jain *et al.*, 2003 ▸). VapA is a surface-attached 17 kDa protein that is released into the phagosome during infection and that increases the permeability of phagosome and lysosome membranes to some ions and protons (von Bargen *et al.*, 2019 ▸). The critical activity of VapA is the collapse of the pH gradient across the phagosome membrane, as chemical pH neutralization of the endocytic and phagocytic continuum using any of several pH-neutralizing drugs enables virulence-plasmid-deficient (avirulent) *R. equi* to multiply in macrophages. Interestingly, a proteinase K-digested version of VapA, which corresponds to the VapB fragment in this study, still has membrane-permeabilizing activity and supports intracellular multiplication of plasmid-less *R. equi* (von Bargen *et al.*, 2019 ▸).

Although the membrane-binding and permeabilizing activities of VapA have been clearly documented (Wright *et al.*, 2018 ▸; von Bargen *et al.*, 2019 ▸), it is not known how VapA exerts its effect on membranes. The same applies to virulence-associated protein B (VapB), which is also encoded on a virulence plasmid; this plasmid is mostly absent from foal isolates and is predominantly found in porcine *R. equi* isolates (Ribeiro *et al.*, 2011 ▸; Letek *et al.*, 2008 ▸). Recent data suggest that VapB, although 78% identical to VapA in its amino-acid sequence, is not required for the virulence of VapB-producing *R. equi* strains (Willingham-Lane *et al.*, 2018 ▸). It has been reported that VapB binds more weakly to yeast plasma membranes and to some liposomes than VapA does (Wright *et al.*, 2018 ▸). However, VapB does bind well to some membranes (A. Haas. P. Hansen & J. Kniewel, unpublished data), possibly depending on their precise composition.

We reasoned that in the absence of a VapA structure and given the very high level of sequence identity (78%) of VapB to VapA, solving the structure of VapB could hint at possible membrane-interacting surfaces, which then could provide clues as to how VapA exerts its pathogenic effects (Geerds *et al.*, 2014 ▸). Shortly after our VapB structure, the very similar structures of *R. equi* VapD and VapG were published (Whittingham *et al.*, 2014 ▸; Okoko *et al.*, 2015 ▸). Vap proteins consist of an N-terminal region that is variable in sequence and is probably intrinsically unstructured and a stably folded C-terminal core with high sequence conservation. The protease-resistant core of *R. equi* Vap proteins forms an eight-stranded β-barrel consisting of two Greek-key motifs. Vaps have internal symmetry, with a pseudo-twofold axis relating the two Greek-key motifs, suggesting that the structure arose by gene duplication (Whittingham *et al.*, 2014 ▸). A short helix connects the two half-barrels, resulting in an unusual topology that is very different from the antiparallel all-next-neighbour topology typically found in (eight-stranded) β-barrels. We term the end of the barrel containing the α-helix and both termini the bottom. The top end contains the four connections between odd- and even-numbered strands, two of which are next neighbours (β1–β2 and β5–β6) and the other two of which are crossover connections (β3–β4 and β7–β8).

The Vap surface contains a flat region devoid of side chains due to the accumulation of glycines. In the VapD structure, two octyl-β-d-glucoside molecules from the crystallization solution bind to this ‘bald’ spot that may allow *R. equi* Vaps to interact with large nonpolar surfaces (Whittingham *et al.*, 2014 ▸). We had suggested a functional similarity between Vaps and avidins, which also form eight-stranded β-barrels (Geerds *et al.*, 2014 ▸). However, avidins have a different, all-next-neighbour topology. Avidins bind biotin in a groove at the top of the molecule (Livnah *et al.*, 1993 ▸; Weber *et al.*, 1989 ▸). Whittingham and coworkers also noted the structural similarity between VapD and bradavidin 2, an avidin-like protein from *Bradyrhizobium japonicum* (Leppiniemi *et al.*, 2013 ▸), but suggested that VapD is not involved in small-molecule binding due to the absence of a cavity (Whittingham *et al.*, 2014 ▸). Okoko and coworkers found no significant depressions on the surfaces of VapB, VapD and VapG that would indicate the presence of a ligand-binding cavity or an enzyme active site (Okoko *et al.*, 2015 ▸). Moreover, Okoko and coworkers noted that avidins lack crossover strands due to their antiparallel all-next-neighbour topology. As a result, the avidin barrels have intrinsically more open structures that allow one end of the barrel to develop into a ligand-binding cavity, in which the barrel interior forms the base. In contrast, Vap proteins have less potential for a binding site due to the crossover connections linking strands β3 to β4 and β7 to β8 at the top and the α-helix that seals the bottom (Okoko *et al.*, 2015 ▸). Nevertheless, experimental data show that *R. equi* Vaps can interact with component(s) of the host cell, most notably membranes. Here, we present a new structure of VapB that highlights a potential binding site for a putative host cell-derived ligand.

## 2. Materials and methods   

### 2.1. Macromolecule production   

VapB was expressed and purified as described by Geerds *et al.* (2014 ▸), with some differences in the digestion with proteinase K. 50 mg VapB was digested with proteinase K (Roth, product key 7528.1) at a 25:1 protein:protease ratio in 50 ml phosphate-buffered saline for 4 h at 37°C. The protease was inactivated by adding phenylmethylsulfonyl fluoride (PMSF) to a final concentration of 2 m*M* and incubating on ice for 30 min. The solution was then dialyzed against 2 l 25 m*M* Tris pH 7, 20 m*M* NaCl, 0.1 m*M* PMSF overnight, with a protein recovery of about 50% (25.4 mg). The proteinase K digest resulted in a single VapB band on SDS–PAGE, in contrast to a previous proteinase K digest, where a double band was observed (Geerds *et al.*, 2014 ▸). Proteinase K-digested VapB was further purified by anion-exchange chromatography on Source Q (GE Healthcare) resin equilibrated in 25 m*M* Tris pH 7, 20 m*M* NaCl. Elution with a salt gradient up to 1 *M* NaCl resulted in a broad and nonsymmetric peak, indicating some heterogeneity. Late peak fractions were pooled, dialyzed against 25 m*M* Tris pH 7.0, 20 m*M* NaCl overnight, concentrated to 16.8 mg ml^−1^ using Vivaspin concentrators, frozen in small aliquots and stored at −80°C. The recovery after proteinase K digestion and purification was about 5% (2.4 mg). Macromolecule-production information is summarized in Table 1 ▸.

### 2.2. Crystallization   

Initial crystals grew in condition F4 of The JCSG Core I Suite (20% PEG 3350, 0.2 *M* magnesium nitrate). The crystal used for data collection grew in a sitting-drop vapour-diffusion setup under optimized conditions (14% PEG 4000, 0.2 *M* magnesium nitrate) with 10 mg ml^−1^ protein in 25 m*M* Tris pH 7,0, 20 m*M* NaCl at 20°C using 1 µl protein solution plus 0.5 µl reservoir solution. A drop ratio of 2:1 protein:precipitant solution has been shown to yield more crystallization hits than a ratio of 1:1 or 1:2 (Ng *et al.*, 2016 ▸), and we routinely use 2:1 drops (Niemann *et al.*, 2006 ▸; Thoms *et al.*, 2011 ▸; Schreiner & Niemann, 2012 ▸; Barden *et al.*, 2014 ▸; Moritzer & Niemann, 2019 ▸; Meyer *et al.*, 2020 ▸). The crystal grew to final dimensions of about 120 × 70 µm in about three weeks. The crystal was cryoprotected in 18% PEG 4000, 0.2 *M* magnesium nitrate, 20% glycerol and flash-cooled in liquid nitrogen. Crystallization information is summarized in Table 2 ▸.

### 2.3. Data collection and processing   

Data were collected on beamline P14 operated by EMBL Hamburg at the PETRA III storage ring, DESY, Hamburg, Germany during setup of this beamline (on 5 October 2012) with an unfocused beam, an aperture size of 150 µm and a fixed wavelength of 1.23953 Å. The beamline was equipped with a vertical ‘hanging’ spindle and a PILATUS 6M detector. Data were collected in two runs. After a short sweep (316 images), the detector distance was decreased to achieve higher resolution. Data were indexed and integrated with *XDS* (Kabsch, 2010 ▸) and both sweeps were scaled together with *XSCALE* using zero-dose extrapolation (Diederichs *et al.*, 2003 ▸). Data-collection and processing statistics are summarized in Table 3 ▸.

### 2.4. Structure solution and refinement   

The structure was solved by molecular replacement in *Phaser* (McCoy *et al.*, 2007 ▸) using a preliminary model of the published *P*6_1_22 structure (Geerds *et al.*, 2014 ▸) as a search model. The preliminary *P*6_1_22 structure had been refined against 1.9 Å resolution home-source data before the high-resolution synchrotron data for the *P*6_1_22 crystal form became available. Two copies of VapB could easily be located and the structure was manually rebuilt in *Coot* (Casañal *et al.*, 2020 ▸) and refined in *REFMAC*5 (Murshudov *et al.*, 2011 ▸) to facilitate quick cycles of building and refinement. Final refinements were performed in *phenix.refine* with TLS refinement enabled (Liebschner *et al.*, 2019 ▸), yielding lower *R* factors. Figures were generated with *PyMOL*. Refinement statistics are summarized in Table 4 ▸.

## 3. Results and discussion   

Here, we determined the structure of the protease-resistant VapB core in a new crystal form with two molecules in the asymmetric unit. The crystals diffracted to 1.71 Å resolution and the structure was solved by molecular replacement. Electron density is visible for residues Gln86–Trp194, while three additional C-terminal residues were resolved in the published VapB structure (Geerds *et al.*, 2014 ▸). Here, we used a different batch of protein that may chemically differ from the previously published batch due to differences in the proteinase K digestion (see Section 2) that resulted in different behaviour of the protein in SDS–PAGE and ion-exchange chromatography. The crystallization conditions also differ from the published conditions, which did not reproducibly yield crystals. It is unclear whether the proteinase K digestion in this work removed the C-terminal residues that were previously visible or whether these residues are present but disordered in the new structure.

**Figure 1:**
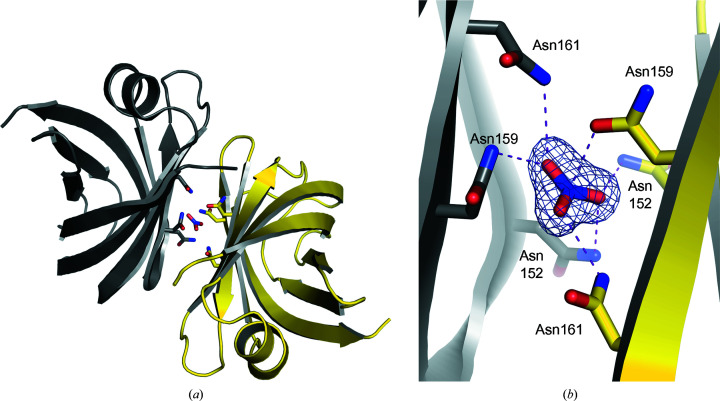
A highly coordinated nitrate ion from the crystallization cocktail bridges two molecules in the asymmetric unit. (*a*) View roughly along the pseudo-twofold axis. Chain *A* is depicted in grey and chain *B* is in yellow. The side chains of Asn152, Asn159 and Asn161 are shown as sticks. (*b*) Dashed purple lines represent polar contacts between the nitrate ion (residue B201) and Asn152, Asn159 and Asn161 from chains *A* (grey) and *B* (yellow). A simulated-annealing OMIT 2*mF*_o_ − *DF*_c_ map is contoured at 1σ around the nitrate ion.

The two monomers in the asymmetric unit arrange around a pseudo-twofold axis (rotation of ∼176°, translation of ∼3 Å; Fig. 1 ▸ *a*). A nitrate ion from the crystallization cocktail, which contained 200 m*M* magnesium nitrate, binds between strands β5 and β6 and is very well defined in the electron density (Fig. 1 ▸ *b*). The same three residues (Asn152, Asn159 and Asn161) from both chains contribute to nitrate binding. The position and orientation of the nitrate relative to the protein is similar but not identical in chains *A* and *B*. The conformation of the asparagine side chains also differs between chains *A* and *B*, particularly for Asn159, which coordinates a nitrate O atom via its amide NH_2_ in chain *A*, while in chain *B* it coordinates the positively charged nitrate N atom via its amide O atom. It appears unlikely that the bound nitrate has any biological relevance. Likewise, we consider the dimeric arrangement to be a mere crystal-packing contact for several reasons: (i) it does not have *C*_2_ point-group symmetry as the vast majority of biological homodimers do (Schulz, 2010 ▸), (ii) it is not predicted to be stable in solution by the *PISA* server (Krissinel & Henrick, 2007 ▸), (iii) it is not present in any of the other Vap structures, even when considering all crystallographic symmetry, and (iv) VapG behaves as monomer in solution, as shown by multi-angle laser light scattering (Whittingham *et al.*, 2014 ▸).

**Figure 2:**
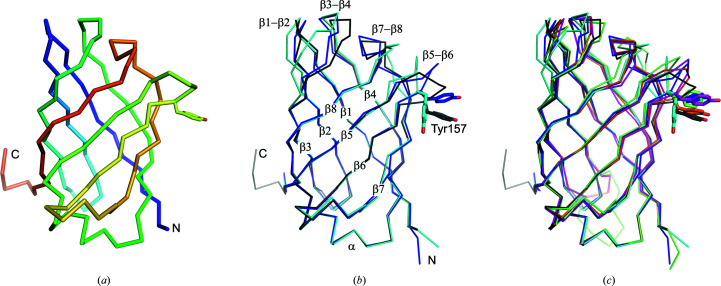
Overlay of *R. equi* Vap structures. (*a*) The previously published VapB structure with PDB code 4cv7 is coloured blue to red from the N-terminus to the C-­terminus in order to illustrate the complex topology of the Vap β-barrel. (*b*) Overlay of three VapB structures: PDB entry 4cv7 in grey, PDB entry 7b1z chain *A* in cyan and PDB entry 7b1z chain *B* in blue. (*c*) Overlay of all published structures of *R. equi* Vaps: VapB, PDB entry 4cv7, grey; VapB, PDB entry 7b1z, chain *A*, cyan; VapB, PDB entry 7b1z, chain *B*, blue; VapD, PDB entry 4csb, lime; VapG, PDB entry 5aeo, chain *A*, orange; VapG, PDB entry 5aeo, chain *B*, magenta.

**Figure 3:**
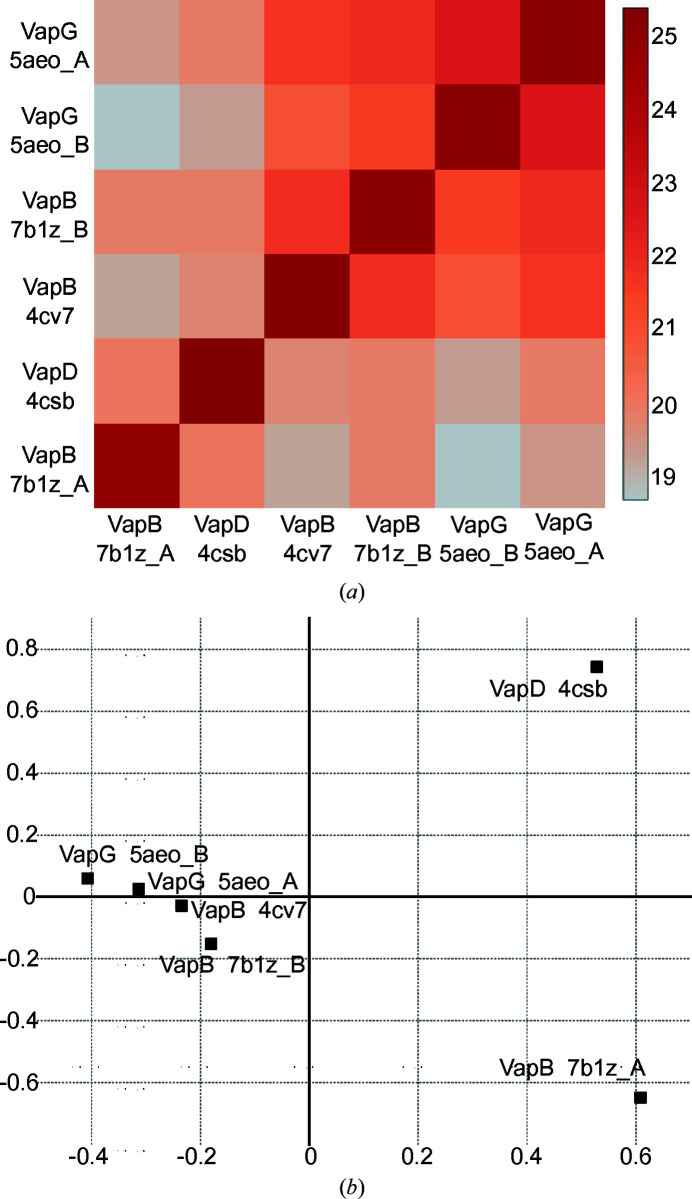
Structural comparison of loop regions at the top of the Vap β-barrel. An all-against-all analysis was performed with the *DALI* server (Holm, 2020 ▸) for truncated Vap structures, in which flexible (N- and C-termini) or variable (β2–β3 loop) residues had been removed. These six truncated Vap structures correspond to VapB residues 89–108 and 113–192. (*a*) Heat map of pairwise *Z*-scores. The bar on the right assigns colours to *Z*-­scores. The pairwise root-mean-square distances (r.m.s.d.s) are given in Supplementary Table S1. (*b*) VapD and the open VapB structure appear as outliers in correspondence analysis, a multidimensional scaling method that positions data points with the most similar structural neighbourhoods near each other. The horizontal and vertical axes represent the first and the second eigenvector, respectively.

**Figure 4:**
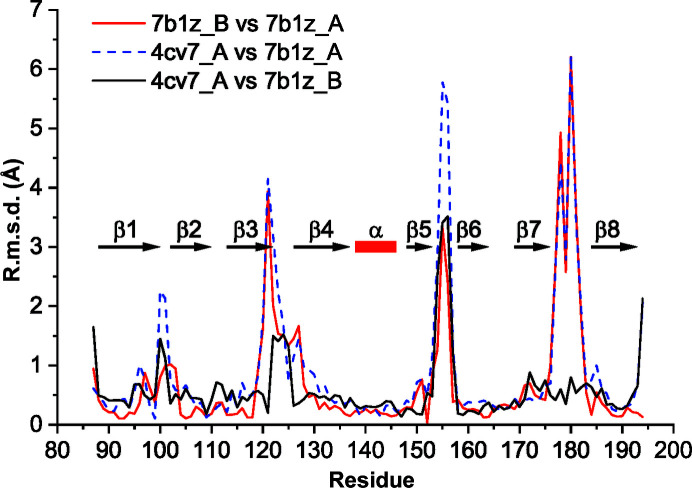
Pairwise comparison of all VapB structures. The per-residue r.m.s.d. of C^α^ atoms was calculated with *LSQKAB* (Kabsch, 1976 ▸). The position of secondary-structure elements is indicated for the first VapB structure (PDB entry 4cv7).

**Figure 5:**
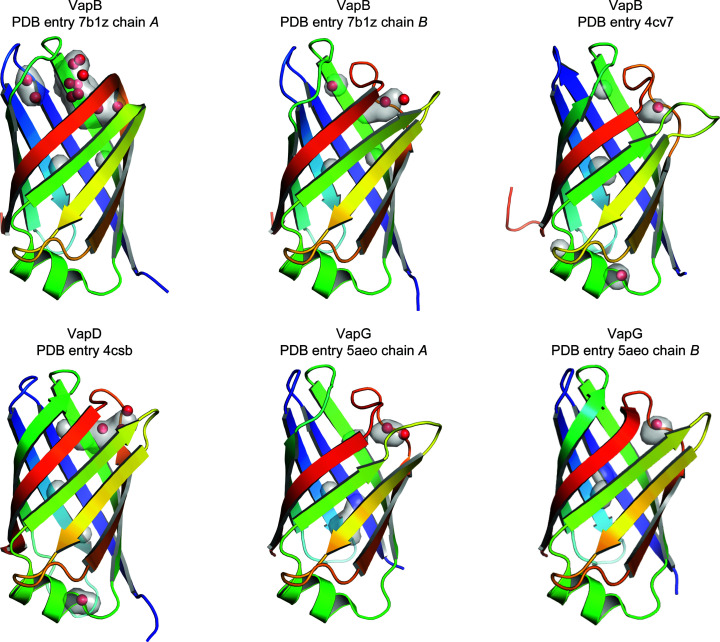
Cavities in Vap structures. All Vaps were structurally aligned. The cartoon representation is coloured blue to red from the N-terminus to the C-terminus. The setting ‘Cavities and Pockets Only’ in the surface representation of *PyMOL* was used to display completely enclosed and surface-accessible cavities as a transparent grey surface. Water molecules located completely or partially within these pockets are shown as red spheres. Only chain *A* of the new VapB structure (PDB entry 7b1z chain *A*) has a large, water-filled pocket between the β3–β4 loop and the β7–β8 loop.

**Figure 6:**
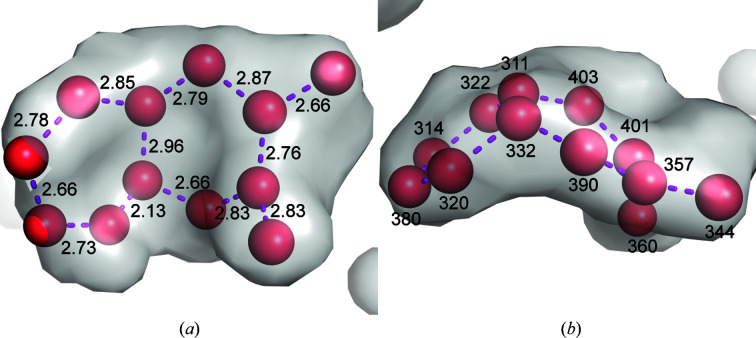
Water molecules within the large VapB pocket. The two views are rotated by 90° relative to each other. (*a*) Distances given in Å are shown as purple dashed lines. (*b*) The residue numbering in PDB entry 7b1z chain *A* is indicated in black. Waters 311 and 322, which are only 2.13 Å apart, are modelled with partial occupancies of 0.5.

An overlay of all three VapB monomers reveals that the bottom of the barrel is structurally largely invariant, while the loops at the top are flexible (Figs. 2 ▸ *a* and 2 ▸ *b*). An extended comparison of all available Vap structures, including VapD and VapG (Fig. 2 ▸ *c*), shows that the structural conservation of the bottom end extends across the Vap family, with the exception of the β2–β3 loop, which differs in conformation between VapB and VapG and harbours a five-residue insertion in VapD. The conformation of the loops at the top of the barrel apparently strongly depends on the crystal-packing environment. The loops at the top of the barrel are involved in crystal-packing contacts that differ between the three crystallographically independent VapB molecules (Supplementary Fig. S1). An all-against-all analysis of the available Vap structures using the *DALI* server (Holm, 2020 ▸) revealed that with regard to these loops, chain *A* of our new VapB structure is most distant from the other Vap structures. Two of the three VapB structures (PDB entries 4cv7 and 7b1z chain *B*) cluster with the two VapG structures (PDB entries 5aeo chain *A* and 5aeo chain *B*). VapD (PDB entry 4csb) and VapB (PDB entry 7b1z chain *A*) are distinct from the VapG/VapB cluster and from each other (Fig. 3 ▸). The side chain of Tyr157 also adopts a unique rotamer in PDB entry 7b1z chain *A* (Fig. 2 ▸). In the first VapB structure the loops at the top of the barrel were suggested to be flexible because of their high *B* factors. Moreover, the β1–β2 and the β7–β8 loops were modelled with double conformations (Geerds *et al.*, 2014 ▸). A pairwise comparison of all three VapB structures shows that the largest backbone displacements of chain *A* of our new structure (PDB entry 7b1z chain *A*) compared with the first VapB structure (PDB entry 4cv7) or chain *B* of the new VapB structure (PDB entry 7b1z chain *B*) occur in the β7–β8 loop, the β5–β6 loop and the β3–β4 loop (Fig. 4 ▸). In contrast, the β7–β8 loop has almost identical conformations in PDB entries 4cv7 and 7b1z chain *B*. This is reminiscent of streptavidin, in which loops connecting the eight β-strands and especially the flexible binding loop can adopt different conformations either depending on the state (unbound versus biotin-bound) or as a consequence of crystal-packing interactions (Freitag *et al.*, 1997 ▸; Le Trong *et al.*, 2011 ▸). Compared with the other Vap structures, the β3–β4 loop and the β7–β8 loop of PDB entry 7b1z chain *A* move outwards in opposite directions, creating a pocket between them. Okoko and coworkers suggested that Vap proteins may have functions that only manifest upon the conformational changes that frequently accompany the binding of small molecules to proteins (Okoko *et al.*, 2015 ▸). Our new VapB structure reveals that despite the crossover connections, VapB can form a large cavity between the β3–β4 loop and the β7–β8 loop (Fig. 5 ▸). In all other Vap structures smaller cavities are present between the β5–β6 loop and the β7–β8 loop. The bottom of the large VapB cavity is formed by hydrophobic residues (Val96, Phe105, Phe130, Ala153, Leu158 and Ala177), while its side and mouth contain both hydrophobic (for example Ile124 and Val181) and polar (for example Ser98, Gln103, Thr123, Tyr151 and Ser179) residues. The large cavity in VapB contains 12 ordered water molecules (A311, A314, A320, A322, A332, A344, A357, A360, A380, A390, A401 and A403), giving a hint on the size and shape of a putative ligand (Fig. 6 ▸). An overlay of VapB and bradavidin 2 in complex with biotin (PDB entry 4ggz; Leppiniemi *et al.*, 2013 ▸) using the *TopMatch* server (Wiederstein & Sippl, 2020 ▸) places the biotin-binding site of bradavidin 2 in roughly the same position as the cavity of VapB (Supplementary Fig. S2).

In summary, our new VapB structure suggests that the helix-capped bottom of the Vap β-barrel is structurally highly conserved and very rigid, whereas the top end is flexible. A conformational change of the loops at the top end of the VapB barrel opens a cavity that might act as binding site for a ligand. Uncovering the identity of a putative Vap ligand would substantially advance our understanding of how Vaps exert their virulence function during *R. equi* infections.

## Supplementary Material

PDB reference: VapB from the intracellular pathogen *Rhodococcus equi*, 7b1z

Supplementary Table and Figures. DOI: 10.1107/S2053230X2100738X/ow5027sup1.pdf

## Figures and Tables

**Table 1:** Macromolecule-production information

Source organism	*Rhodococcus equi* strain PAM 1593
DNA source	Sequenced virulence plasmid
Forward primer†	**CAT CAT CAC CAC CAT CAC** *GTG CTG GAT TCC GGA GGC GGC* (His His His His His His Val36 Leu37 Asp38 Ser39 Gly Gly Gly)
Reverse primer‡	GTG GC|G GCC GCT CTA **TTA TTA** *TGC AAC CTC CCA GTT GTG* (His Asn Trp Glu Val Ala197 Stop Stop)
Expression vector	pETite N-His Kan (BioCat, Heidelberg, Germany)
Expression host	*E. coli* BL21 (DE3)
Complete amino-acid sequence of the construct produced§	M**HHHHHH**VLDSGGGSALLKDGAGSGEVGSQAYDSSTVSSNLQKAETNGPVGLAGTAEQEQQYDVHGNVISAAVYQKFHVYGPEDMVFDGDAGGLTIPGAGAFWGTLFTSDLQRLYKDTVSFQYNALGTYLNINFFDSSGGFLGHIQAGAVSAVVGVGGGSGSWHNWEVA

† The codons for the hexahistidine tag are in bold and the sequence coding for VapB starting at Val36 is in italics.

‡ The sequence coding for VapB is in italics, two stop codons are in bold, the NotI restriction site is underlined and the cleavage site of NotI is indicated by a vertical line.

§ The expressed construct comprises residues Val36–Ala197 of VapB. The additional hexahistidine tag is shown in bold.

**Table 2:** Crystallization

Method	Vapour diffusion
Plate type	Cryschem M 24-well sitting drop
Temperature (K)	293
Protein concentration (mg ml^−1^)	10
Buffer composition of protein solution	25 m*M* Tris pH 7.0, 20 m*M* NaCl
Composition of reservoir solution	14% PEG 4000, 0.2 *M* Mg(NO_3_)_2_
Volume and ratio of drop	1 µl protein solution + 0.5 µl reservoir solution
Volume of reservoir (µl)	500

**Table 3:** Data collection and processing Values in parentheses are for the highest resolution shell.

Diffraction source	EMBL beamline P14 (unfocused beam), PETRA III, DESY
Wavelength (Å)	1.23953
Temperature (K)	100
Detector	PILATUS 6M
Crystal-to-detector distance (mm)	First sweep, 290; second sweep, 243
Rotation range per image (°)	0.1
Total rotation range (°)	First sweep, 31.6; second sweep, 180.4
Exposure time per image (s)	1
Space group	*C*222_1_
*a*, *b*, *c* (Å)	56.94, 65.34, 124.26
Mosaicity (°)	0.12
Resolution range (Å)	50–1.71 (1.75–1.71)
Total No. of reflections	178471 (8435)
No. of unique reflections	25467 (1845)
Completeness (%)	99.8 (99.3)
Multiplicity	7.0 (4.6)
〈*I*/σ(*I*)〉	28.7 (2.8)
CC_1/2_ (%)	100 (85.0)
*R*_meas_ (%)	3.9 (65.7)
Overall *B* factor from Wilson plot (Å^2^)	33

**Table 4:** Structure solution and refinement Values in parentheses are for the highest resolution shell.

Resolution range (Å)	42.93–1.71 (1.78–1.71)
Completeness (%)	99.8 (99)
No. of reflections, working set	25424 (2640)
No. of reflections, test set	1265 (133)
Final *R*_work_	0.1539 (0.1816)
Final *R*_free_	0.1816 (0.2997)
No. of non-H atoms	
Protein	1733
Water	184
Other (nitrate, glycerol)	38
Total	1955
R.m.s. deviations	
Bonds (Å)	0.009
Angles (°)	0.910
Average *B* factors (Å^2^)	
Protein	30.4
Water	38.4
Other (nitrate, glycerol)	47.1
Ramachandran plot	
Most favoured (%)	97.20
Allowed (%)	2.80
