# Supplementary material for: Conformational changes of loops highlight a potential binding site in Rhodococcus equi VapB
Source: Acta Crystallogr F Struct Biol Commun. 2021 Jul 28;77(Pt 8):246–53. doi: 10.1107/S2053230X2100738X (PMC8329714; doi:10.1107/S2053230X2100738X)
Supplement: Supplementary file 1 [file f-77-00246-sup1.pdf]

STRUCTURAL BIOLOGY
COMMUNICATIONS

Volume 77 (2021)

Supporting information for article:

Conformational changes of loops highlight a potential binding site in *Rhodococcus equi* VapB

Christina Geerds, Albert Haas and Hartmut H. Niemann

Table S1 Pairwise root mean square distance (rmsd) values of truncated Vaps as supporting information for Figure 3 that depicts the structural comparison of loop regions at the top of the Vap β-barrel

An all-against-all analysis was performed with the DALI server (Holm, 2020) for truncated Vap structures, in which flexible (N- and C-termini) or variable (β2-β3 loop) residues had been removed. These six truncated Vap structures correspond to VapB residues 89-108 and 113-192. Rmsd values are those reported in the Summaries tab of the DALI results.

	**VapB 4cv7**	**VapB 7b1z_A**	**VapB 7b1z_B**	**VapD 4csb**	**VapG 5aeo_A**	**VapG 5aeo_A**
**VapB 4cv7**	0.0	1.4	0.8	1.3	0.7	0.9
**VapB 7b1z_A**		0.0	1.1	1.1	1.3	1.4
**VapB 7b1z_B**			0.0	1.2	0.6	0.7
**VapD 4csb**				0.0	1.3	1.2
**VapG 5aeo_A**					0.0	0.5
**VapG 5aeo_A**						0.0

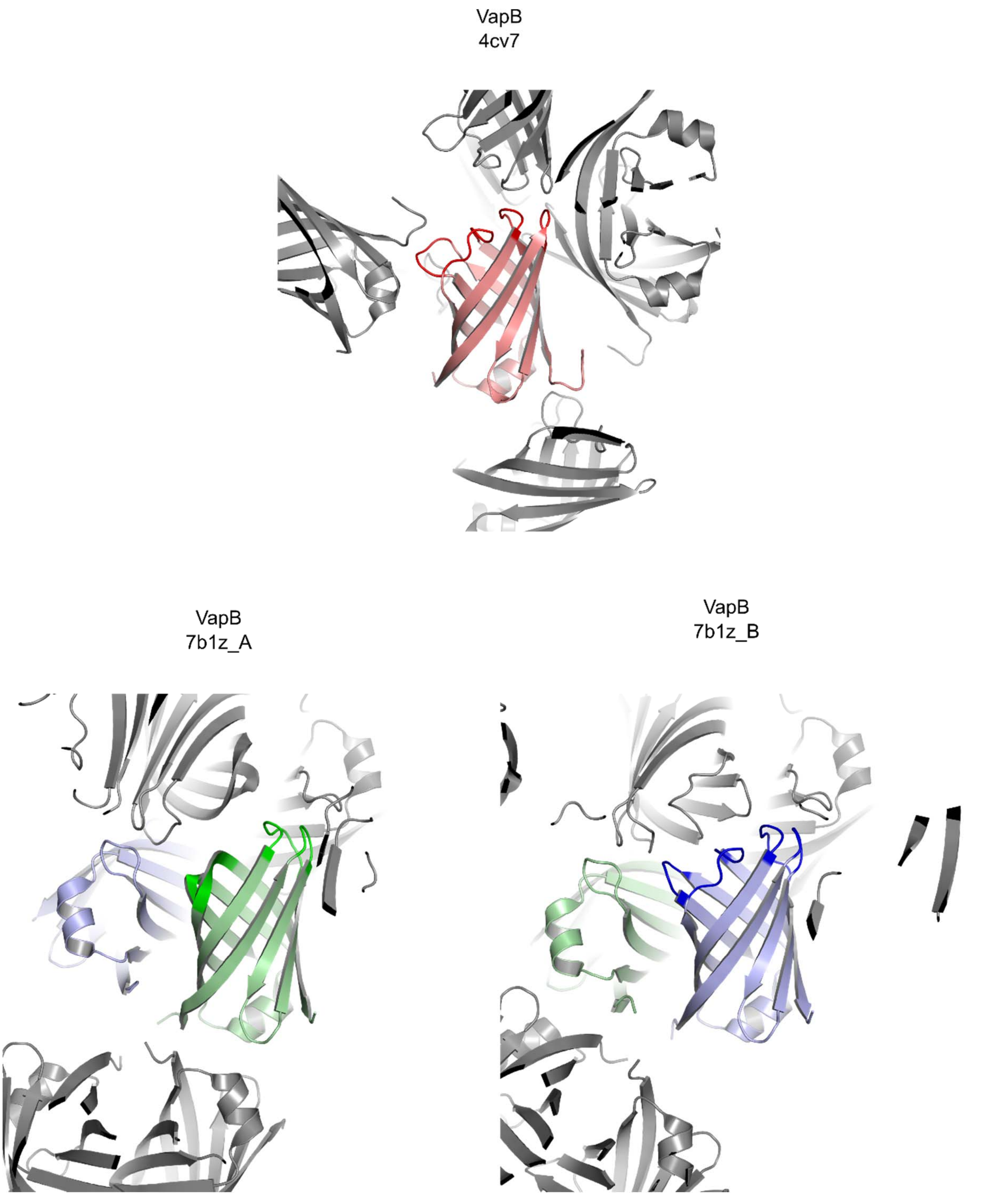

Figure S1 Crystal packing contacts of loop regions at the top of the VapB barrel. All three crystallographically independent VapB chains are shown in the same orientation. Chain A of PDB ID 4cv7 is coloured in salmon, chain A of PDB ID 7b1z is shown in palegreen and chain B of PDB ID 7b1z is shown in lightblue. The loop regions at the top of the VapB barrel are highlighted in red, green and blue. Symmetry-related molecules are shown in grey. The loops at the top of the VapB barrel are involved in different crystal contacts in each chain. In the new VapB structure (7b1z), the loops of chain A contact the loop region of a symmetry-related chain A, while the loops of chain B contact the loop region of a symmetry-related chain B.

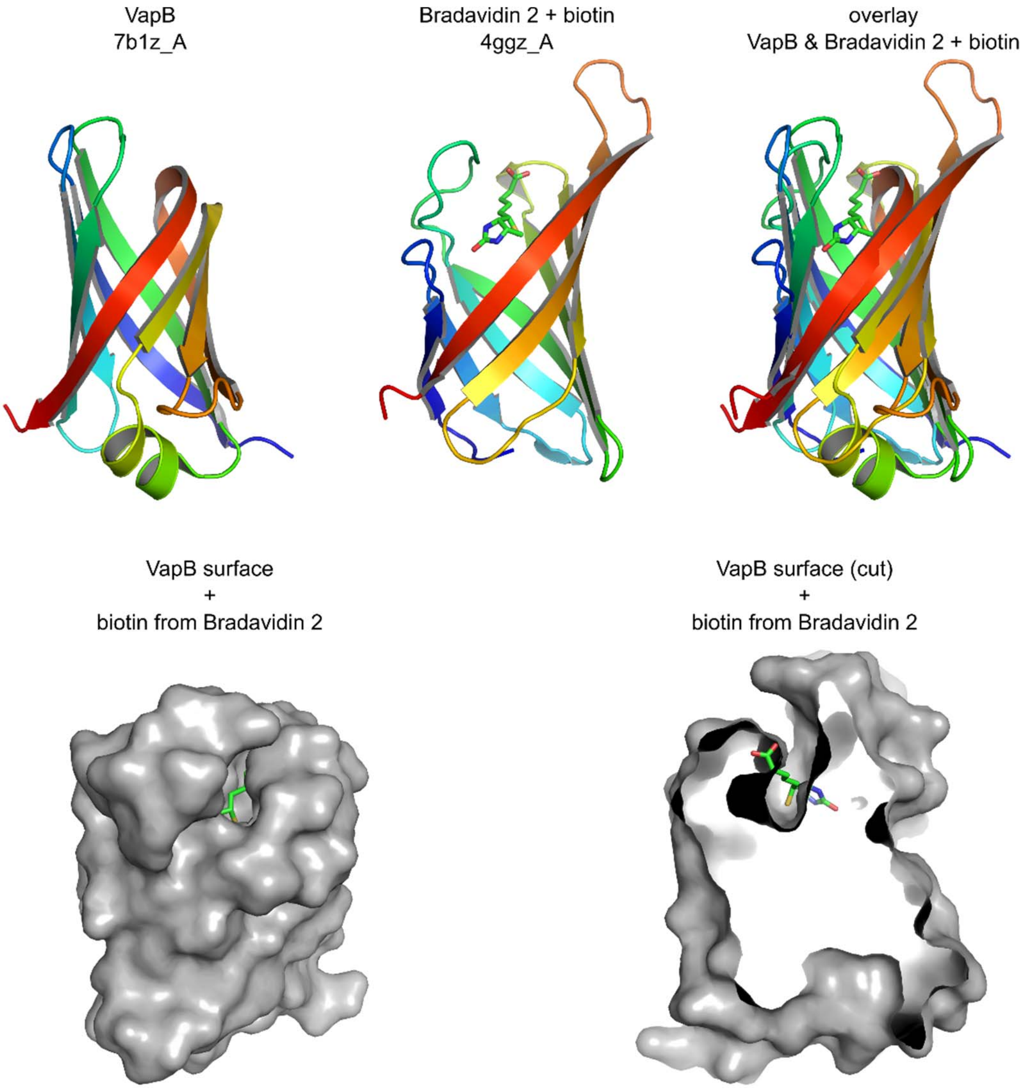

Figure S2 The cavity of VapB is located in roughly the same place as the biotin binding site of Bradavidin 2 (Leppiniemi *et al.*, 2013). The overlay of VapB and Bradavidin 2 was generated with the TopMatch server (Wiederstein & Sippl, 2020) The cartoon representation is coloured blue to red from N- to C-terminus. Biotin is shown as sticks with green carbon atoms. In the bottom row, biotin from Bradavidin 2 is overlaid with a grey surface representation of VapB. A part of the biotin is visible within the VapB cavity.

References

Holm, L. (2020). *Protein Sci.* **29**, 128–140.

Leppiniemi, J., Meir, A., Kahkonen, N., Kukkurainen, S., Maatta, J. A., Ojanen, M., Janis, J., Kulomaa, M. S., Livnah, O. & Hytonen, V. P. (2013). *Protein Sci.* **22**, 980–994.

Wiederstein, M. & Sippl, M. J. (2020). *Nucleic Acids Res.* **48**, W31–W35.
